# Evaluation of Hypertensive Disorder of Pregnancy and High Refractive Error in Offspring During Childhood and Adolescence

**DOI:** 10.1001/jamanetworkopen.2023.8694

**Published:** 2023-04-18

**Authors:** Meiyan Li, Chen Huang, Weiming Yang, Priscilla Ming Yi Lee, Yahang Liu, Ruilang Lin, Guoyou Qin, Yongfu Yu, Xingtao Zhou, Jiong Li

**Affiliations:** 1 Department of Ophthalmology, Eye & ENT Hospital of Fudan University, Key Lab of Myopia, Ministry of Health, Shanghai, China; 2 Department of Biostatistics, School of Public Health, The Key Laboratory of Public Health Safety of Ministry of Education, Fudan University, Shanghai, China; 3 Department of Ophthalmology, Children’s Hospital of Fudan University, National Children's Medical Center, Shanghai, China; 4 Department of Clinical Medicine, Department of Clinical Epidemiology, Aarhus University, Aarhus, Denmark; 5 Shanghai Institute of Infectious Disease and Biosecurity, Shanghai, China; 6 State Key Laboratory of Reproductive Medicine, Nanjing Medical University, Nanjing, China; 7 Department of Epidemiology, School of Public Health, Nanjing Medical University, Nanjing, China

## Abstract

**Importance:**

Growing evidence indicates that adverse prenatal or intrauterine environments might contribute to the development of high refractive error (RE) later in life. However, the association of maternal hypertensive disorder of pregnancy (HDP) with high RE in offspring during childhood and adolescence remains unknown.

**Objective:**

To investigate the association between maternal HDP and overall and type-specific high REs in offspring in childhood and adolescence.

**Design, Setting, and Participants:**

This nationwide population-based cohort study included live-born individuals born in Denmark from 1978 to 2018 in the Danish national health registers. Follow-up started at the date of birth and ended at the date of RE diagnosis, 18th birthday, death, emigration, or December 31, 2018, whichever came first. Data analyses were conducted from November 12, 2021, through June 30, 2022.

**Exposures:**

Maternal HDP (n = 104 952), including preeclampsia or eclampsia (n = 70 465) and hypertension (n = 34 487).

**Main Outcomes and Measures:**

The main outcomes were the first occurrence of high RE (hyperopia, myopia, and astigmatism) in offspring. A Cox proportional hazards regression model was used to examine the association between maternal HDP and risk of high RE in offspring from birth until age 18 years, adjusting for multiple potential confounders.

**Results:**

This study included 2 537 421 live-born individuals, 51.30% of whom were male. During the follow-up of up to 18 years, 946 offspring of 104 952 mothers with HDP (0.90%) and 15 559 offspring of 2 432 469 mothers without HDP (0.64%) were diagnosed with high RE. The cumulative incidence of high RE was higher in the exposed cohort (1.12%; 95% CI, 1.05%-1.19%) than in the unexposed cohort (0.80%; 95% CI, 0.78%-0.81%) at 18 years of age (difference: 0.32%; 95% CI, 0.25%-0.40%). Offspring born to mothers with HDP had a 39% increased risk of overall high RE (hazard ratio [HR], 1.39; 95% CI, 1.31-1.49). Sibling-matched analysis revealed an increased risk of overall high RE in half siblings (HR, 1.21; 95% CI, 1.05-1.39) and full siblings (HR, 1.15; 95% CI, 0.99-1.34), but the difference was not significant for the latter. The elevated risks were observed for hypermetropia (HR, 1.41; 95% CI, 1.30-1.52), myopia (HR, 1.30; 95% CI, 1.10-1.53), and astigmatism (HR, 1.45; 95% CI, 1.22-1.71). The increased risk of high RE persisted among offspring aged 0 to 6 years (HR, 1.51, 95% CI, 1.38-1.65), 7 to 12 years (HR, 1.28; 95% CI, 1.11-1.47), and 13 to 18 years (HR, 1.16; 95% CI, 0.95-1.41), but the difference was not significant for the oldest group. When considering both timing of diagnosis and severity of maternal preeclampsia, the highest risk was observed in offspring prenatally exposed to early-onset and severe preeclampsia (HR, 2.59; 95% CI, 2.17-3.08).

**Conclusions and Relevance:**

In this cohort study of the Danish population, maternal HDP, especially early-onset and severe preeclampsia, was associated with an increased risk of high RE in offspring during childhood and adolescence. These findings suggest that early and regular RE screening should be recommended for children of mothers with HDP.

## Key Points

**Question:**

Is there an association between maternal hypertensive disorder of pregnancy and high refractive error among offspring during childhood and adolescence?

**Findings:**

In this cohort study of 2 537 421 individuals born in Denmark from 1978 to 2018, offspring born to mothers with hypertensive disorder of pregnancy had a 39% higher estimated risk of overall high refractive error, including hyperopia, myopia, and astigmatism. The elevated risk persisted across different age groups from birth to 12 years, and offspring prenatally exposed to early-onset and severe preeclampsia had the highest risk.

**Meaning:**

The findings suggest that early and regular refractive error screening should be recommended for children of mothers with hypertensive disorder of pregnancy.

## Teaser

This cohort study assesses the association between maternal hypertensive disorder of pregnancy and high refractive error among offspring during childhood and adolescence in Denmark.

## Introduction

Refractive error (RE), defined as defect in the focus of the light on the retina resulting in blurred vision, is the leading cause of visual impairment worldwide.^1,2^ The prevalence of RE among children and adolescents has been increasing in recent decades, and individuals with high RE are particularly prone to functional blindness at a relatively young age.^3,4^ Growing evidence has shown that prenatal adversity, such as maternal gestational diabetes and maternal smoking during pregnancy, is associated with increased risk of overall or type-specific REs in offspring.^5,6,7,8,9,10,11^ These findings indicate that adverse prenatal or intrauterine environments might contribute to the development of high RE later in life.^12^

Hypertensive disorder of pregnancy (HDP) is one of the most common complications during pregnancy, affecting 5% to 10% of pregnancies.^13^ Hypertensive disorder of pregnancy has been shown to be associated with a number of adverse health outcomes in pregnant women and their offspring in both the short and the long term.^14,15,16,17,18^ Previous studies reported that maternal HDP may be associated with abnormal visual development and eye diseases in offspring.^19,20,21,22,23,24^ A case-control study with 77 children showed that mothers of children with congenital myopia were more likely to have hypertension during pregnancy.^19^ However, whether or to what extent prenatal exposure to maternal HDP is associated with increased risk of overall high RE and specific types of RE, including myopia, hyperopia, and astigmatism, in offspring in childhood and adolescence remains unknown. In addition, as the ocular refractive development and the eye-using behaviors change during different age periods in offspring^12^ and the pathophysiological mechanisms may differ by the timing of onset and severity of preeclampsia,^25^ it is necessary to investigate the influence of timing of onset, severity of preeclampsia, and offspring age on the association of maternal HDP with high RE in offspring.

Using data from several nationwide registers in Denmark, we aimed to investigate the association of overall and type-specific maternal HDP with overall and type-specific high REs in offspring in childhood and adolescence. The association of maternal HDP with high RE in different age groups of the offspring was also evaluated. We further examined whether the timing of onset and severity of preeclampsia was associated with higher estimated risk of high RE in offspring.

## Methods

### Study Population

We conducted a population-based cohort study based on the Danish Medical Birth Register, which contains prenatal and birth information on nearly all births in Denmark since its establishment.^26^ A unique personal identification number was assigned to all Danish residents, allowing linkage of information across different registers in Denmark.^26^ We included live births in Denmark between 1978 and 2018. Follow-up started at the date of birth and ended at the date of RE diagnosis, 18th birthday, death, emigration, or December 31, 2018, whichever came first. Individuals who emigrated or died during follow-up were censored at the time of emigration or death. Detailed descriptions of registers used in this study are provided in eTable 1 in Supplement 1. The study was approved by the Data Protection Agency. By Danish law, no informed consent is required for a register-based study based on anonymized data. The study followed the Strengthening the Reporting of Observational Studies in Epidemiology (STROBE) reporting guideline.

### Maternal HDP

Maternal HDP was identified from the Danish National Patient Register and Danish Medical Birth Register using the *International Classification of Diseases, Eighth Revision (ICD-8)* codes from 1978 to 1993 and *International Statistical Classification of Diseases and Related Health Problems, Tenth Revision (ICD-10)* codes from 1994 to 2018 (eTable 2 in Supplement 1).^26^ Maternal HDP included preeclampsia or eclampsia and hypertension. Preeclampsia or eclampsia was further divided into moderate preeclampsia, severe preeclampsia, HELLP (hemolysis, elevated liver enzyme level, and low platelet count) syndrome, unspecified preeclampsia, and eclampsia. Hypertension was categorized into pregestational hypertension and gestational hypertension. For women with multiple HDP diagnoses, we classified HDP according to the disease severity: eclampsia, preeclampsia, pregestational hypertension, and gestational hypertension.

### Outcome of Interest

The primary outcome of interest was the first occurrence of high RE identified in the Danish National Patient Register using the *ICD-8* and *ICD-10* codes.^26^ The secondary outcomes were specific types of high RE in offspring, including hypermetropia, myopia, astigmatism, and other types of high RE. Detailed *ICD-8* and *ICD-10* codes of overall and type-specific high REs are provided in eTable 3 in Supplement 1.

### Covariates

Several potential confounders were selected as covariates using a directed acyclic graph (eFigure 1 in Supplement 1), including sex (male, female), singleton (yes, no), parity (1, 2, or ≥3 children), birth year of the child (1978-1980; 5-year intervals during 1981-2015 and 2016-2018), maternal age (<20, 20-24, 25-29, 30-34, or ≥35 years), maternal smoking during pregnancy (yes, no), maternal cohabitation (single, cohabitating), maternal residence (Copenhagen, city with ≥100 000 inhabitants, or other), maternal country of origin (Denmark, not Denmark), duration of maternal education before pregnancy (0-9, 10-14, or ≥15 years), maternal income at birth (no income, 3 tertiles), maternal prepregnancy body mass index (BMI; calculated as weight in kilograms divided by height in meters squared) (<18.5, 18.5-24.9, 25.0-29.9, or ≥30.0), maternal history of RE before childbirth (yes, no), and paternal history of RE before childbirth (yes, no). Information on maternal and offspring characteristics was retrieved from the Danish National Patient Register, the Danish Medical Birth Register, and the Danish Integrated Database for Labor Market Research.^26,27^ A multiple imputation procedure with fully conditional specification in SAS, version 9.4 (SAS Institute Inc) was used to impute 10 replications (greater than the percentage of data missing) to deal with missing values.^28^ The multiple imputation methods are described in detail in eAppendix 1 in Supplement 1.

### Statistical Analysis

Data analyses were conducted from November 12, 2021, through June 30, 2022. Considering deaths as competing events, we estimated the cumulative incidence of high RE among offspring exposed and unexposed to maternal HDP. A Cox proportional hazards regression model with age as the time scale was used to estimate the hazard ratios (HRs) and 95% CIs of the association between maternal HDP and overall and type-specific high REs in offspring. Robust variance was used to account for the correlations between siblings.^29^ The log-minus-log plot suggested that the proportional hazard assumption was not violated (eFigure 2 in Supplement 1). We evaluated the association of maternal HDP with high RE in different age groups of the offspring (0-6, 7-12, and 13-18 years).^12^ We further examined whether the association between maternal HDP and high RE in offspring differed by the timing of diagnosis and severity of preeclampsia.^25^ For severity, preeclampsia was categorized as moderate and severe (including severe preeclampsia and HELLP syndrome). For timing of diagnosis, preeclampsia was categorized into early onset (diagnosed before 34 weeks’ gestation) and late onset (diagnosed at or after 34 weeks’ gestation).

We performed several sensitivity analyses. To account for the influence of uncontrolled confounding due to the genetic or shared familial characteristics, we conducted a sibship analysis using a stratified Cox proportional hazards regression model by including a stratum for each sibling pair. Half-sibling and full-sibling pairs were defined as offspring from the same mother and offspring from both the same mother and father, respectively. We conducted a stratified analysis by baseline characteristics, including singleton, sex, parity, maternal age, maternal smoking, maternal education duration, maternal cohabitation, maternal residence, maternal country of origin, maternal income, maternal prepregnancy BMI, and parental history of high RE. To evaluate the influence of underlying genetic or familial factors, we considered paternal hypertension before pregnancy as a control exposure and examined the association of paternal hypertension with high RE in offspring. To investigate whether fetal growth restriction influenced the association, we stratified offspring by small for gestational age (defined as infants whose birth weight was below the 10th percentile for infants of the same gestational age, sex, and birth year) or not. We undertook several subgroup analyses: an analysis additionally adjusted for paternal hypertension; analyses restricting to offspring born after 1991, 1994, and 2004 to consider the influence of the change from *ICD-8* to *ICD-10* codes and data availability on confounders; a complete cases analysis; and an analysis restricting to offspring born to primipara. We also performed analysis restricting to term-born infants. All analyses were conducted using SAS, version 9.4 and Stata, version 15.1 (StataCorp LLC).

## Results

The study included 2 537 421 offspring born to 1 302 564 mothers; 48.65% of offspring were female, and 51.30% were male. A total of 104 952 offspring (4.14%) were exposed to maternal HDP (preeclampsia or eclampsia: 2.78%; hypertension: 1.36%). Compared with mothers who did not have HDP, mothers with HDP were more likely to live alone, have a higher prepregnancy BMI, and have a history of RE before childbirth (Table 1).

**Table 1.** Baseline Characteristics According to Offspring Exposure to Maternal HDP

Characteristic	Offspring, No. (%)			
	No HDP (n = 2 432 469)	Preeclampsia or eclampsia (n = 70 465)	Hypertension (n = 34 487)	Total (n = 2 537 421)
Outcome				
No	2 416 910 (99.36)	69 799 (99.05)	34 207 (99.19)	2 520 916 (99.3)
Yes	15 559 (0.64)	666 (0.95)	280 (0.81)	16 505 (0.7)
Singleton				
No	76 476 (3.14)	6 420 (9.11)	1 504 (4.36)	84 400 (3.3)
Yes	2 355 993 (96.86)	64 045 (90.89)	32 983 (95.64)	2 453 021 (96.7)
Sex				
Boy	1 247 135 (51.27)	36 633 (51.99)	17 859 (51.78)	1 301 627 (51.3)
Girl	1 184 003 (48.67)	33 793 (47.96)	16 618 (48.19)	1 234 414 (48.6)
Unknown	1 331 (0.05)	39 (0.06)	10 (0.03)	1 380 (0.1)
Maternal parity				
1	1 081 546 (44.46)	46 281 (65.68)	16 307 (47.28)	1 144 134 (45.1)
2	910 979 (37.45)	16 353 (23.21)	11 645 (33.77)	938 977 (37.0)
≥3	439 944 (18.09)	7 831 (11.11)	6 535 (18.95)	454 310 (17.9)
Maternal age at childbirth, y				
<20	55 607 (2.29)	2 111 (3.00)	321 (0.93)	58 039 (2.3)
20-24	420 422 (17.28)	14 356 (20.37)	3 703 (10.74)	438 481 (17.3)
25-29	883 768 (36.33)	25 175 (35.73)	10 065 (29.18)	919 008 (36.2)
30-34	736 200 (30.27)	18 472 (26.21)	11 557 (33.51)	766 229 (30.2)
≥35	336 472 (13.83)	10 351 (14.69)	8 841 (25.64)	355 664 (14.0)
Maternal smoking during pregnancy^a^				
No	1 331 155 (77.22)	39 723 (80.99)	23 862 (83.90)	1 394 740 (77.4)
Yes	317 090 (18.39)	6 895 (14.06)	3 529 (12.41)	327 514 (18.2)
Unknown	75 647 (4.39)	2 429 (4.95)	1 050 (3.69)	79 126 (4.4)
Maternal education duration at childbirth, y				
0-9	632 432 (26.00)	19 812 (28.12)	6 967 (20.20)	659 211 (26.0)
10-14	1 035 184 (42.56)	31 244 (44.34)	15 297 (44.36)	1 081 725 (42.6)
≥15	721 102 (29.64)	18 594 (26.39)	11 827 (34.29)	751 523 (29.6)
Unknown	43 751 (1.80)	815 (1.16)	396 (1.15)	44 962 (1.8)
Maternal cohabitation at childbirth				
No	1 104 341 (45.40)	36 311 (51.53)	16 079 (46.62)	1 156 731 (45.6)
Yes	1 324 415 (54.45)	34 131 (48.44)	18 398 (53.35)	1 376 944 (54.3)
Unknown	3 713 (0.15)	23 (0.03)	10 (0.03)	3 746 (0.1)
Maternal residence at childbirth				
Copenhagen	281 456 (11.57)	7 884 (11.19)	3 739 (10.84)	293 079 (11.6)
City with ≥100 000 inhabitants	313 767 (12.90)	9 536 (13.53)	5 116 (14.83)	328 419 (12.9)
Other	1 837 246 (75.53)	53 045 (75.28)	25 632 (74.32)	1 915 923 (75.5)
Maternal country of origin				
Denmark	294 956 (12.13)	5 550 (7.88)	2 685 (7.79)	303 191 (11.9)
Not Denmark	2 130 963 (87.60)	64 819 (91.99)	31 762 (92.1)	2 227 544 (87.8)
Unknown	6 550 (0.27)	96 (0.14)	40 (0.12)	6 686 (0.3)
Maternal income				
No income	439 493 (18.98)	10 757 (15.99)	5 133 (15.29)	455 383 (18.8)
Less than the lower tertiles	624 787 (26.99)	19 254 (28.61)	8 207 (24.45)	652 248 (27.0)
Lower and higher tertiles	623 272 (26.92)	19 128 (28.43)	10 173 (30.31)	652 573 (27.0)
More than the higher tertiles	624 967 (27.00)	18 143 (26.96)	10 047 (29.93)	653 157 (27.0)
Unknown	2 552 (0.11)	9 (0.01)	NR^b^	2 565 (0.1)
Prepregnancy maternal BMI^c^				
<18.5	37 185 (4.20)	578 (2.24)	399 (2.02)	38 162 (4.1)
18.5-24.9	525 047 (59.24)	11 697 (45.26)	8 454 (42.87)	545 198 (58.5)
25.0-29.9	175 326 (19.78)	6 554 (25.36)	4 814 (24.41)	186 694 (20.0)
≥30.0	99 384 (11.21)	6 182 (23.92)	5 391 (27.34)	110 957 (11.9)
Unknown	49 372 (5.57)	835 (3.23)	660 (3.35)	50 867 (5.5)
Maternal RE history before childbirth				
No	2 424 213 (99.66)	70 166 (99.58)	34 275 (99.39)	2 528 654 (99.7)
Yes	8 256 (0.34)	299 (0.42)	212 (0.61)	8 767 (0.3)
Paternal RE history before childbirth				
No	2 399 617 (98.65)	69 287 (98.33)	33 915 (98.34)	2 502 819 (98.6)
Yes	7 559 (0.31)	204 (0.29)	136 (0.39)	7 899 (0.3)
Unknown	25 293 (1.04)	974 (1.38)	436 (1.26)	26 703 (1.1)

Abbreviations: BMI, body mass index (calculated as weight in kilograms divided by height in meters squared); HDP, hypertensive disorder of pregnancy; NR, not reported; RE, refractive error.

^a^ Maternal smoking during pregnancy was available in Denmark from 1991 to 2018.

^b^ Not allowed to report when there were fewer than 6 cases due to data protection in Denmark.

^c^ Prepregnancy maternal BMI was available in Denmark from 2004 to 2018.

**Figure 1.**
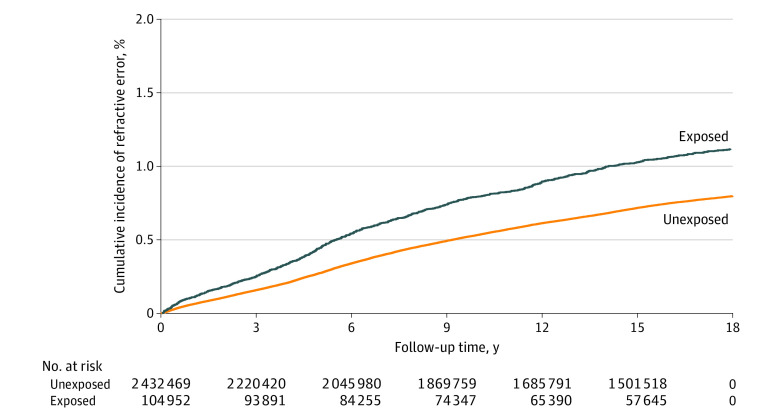
Cumulative Incidence of Overall High Refractive Error Among Offspring Exposed and Unexposed to Maternal Hypertensive Disorder of Pregnancy

During the follow-up of up to 18 years, 946 offspring of 104 952 mothers with HDP (0.90%), including 666 offspring of 70 465 mothers with preeclampsia or eclampsia (0.95%) and 280 offspring of 34 487 mothers with hypertension (0.81%), and 15 559 offspring of 2 432 469 mothers without HDP (0.64%) were diagnosed with high RE (Table 2). Rates of high RE were 0.69 per 1000 person-years for preeclampsia or eclampsia, 0.71 per 1000 person-years for hypertension, and 0.46 per 1000 person-years for no HDP. The cumulative incidence of high RE was higher in the exposed cohort (1.12%; 95% CI, 1.05%-1.19%) compared with the unexposed cohort (0.80%; 95% CI, 0.78%-0.81%) across the follow-up period (difference: 0.32%, 95% CI, 0.25%-0.40%) (Figure 1). Offspring exposed to maternal HDP had a 39% increased risk of overall high RE compared with unexposed offspring (HR, 1.39; 95% CI, 1.31-1.49). The increased risks of high RE associated with exposure to maternal preeclampsia or eclampsia and hypertension were 44% (HR, 1.44; 95% CI, 1.33-1.55) and 31% (HR, 1.31; 95% CI, 1.16-1.47), respectively. Risk increased as the severity of preeclampsia increased (moderate preeclampsia: HR, 1.27 [95% CI, 1.16-1.40]; severe preeclampsia: HR, 1.89 [95% CI, 1.62-2.21]; HELLP syndrome: HR, 2.15 [95% CI, 1.48-3.12]). The patterns were similar for type-specific high REs, with more than 30% increased risk for hypermetropia (HR, 1.41; 95% CI, 1.30-1.52), myopia (HR, 1.30; 95% CI, 1.10-1.53), and astigmatism (HR, 1.45; 95% CI, 1.22-1.71) (Table 2).

**Table 2.** Hazard Ratios for the Association Between Maternal HDP and Overall High RE and Specific Types of High RE in Offspring

Outcome, exposure	Cases, No.	Rate, per 1000 person-years	Hazard ratio (95% CI)	
			Crude	Adjusted^a^
**Overall RE**				
No maternal HDP	15559	0.46	1 [Reference]	1 [Reference]
Maternal HDP				
Overall	946	0.70	1.49 (1.40-1.59)	1.39 (1.31-1.49)
Preeclampsia or eclampsia				
Overall	666	0.69	1.49 (1.38-1.61)	1.44 (1.33-1.55)
Preeclampsia	661	0.69	1.50 (1.38-1.62)	1.44 (1.33-1.56)
Moderate	426	0.59	1.28 (1.16-1.41)	1.27 (1.16-1.40)
Severe	160	1.02	2.18 (1.86-2.54)	1.89 (1.62-2.21)
HELLP syndrome	28	1.55	3.14 (2.16-4.54)	2.15 (1.48-3.12)
Unspecified	47	0.79	1.73 (1.30-2.30)	1.74 (1.31-2.32)
Eclampsia	NR^b^	0.53	1.14 (0.48-2.75)	1.06 (0.44-2.54)
Hypertension				
Overall	280	0.71	1.48 (1.32-1.67)	1.31 (1.16-1.47)
Pregestational hypertension	130	0.81	1.66 (1.40-1.98)	1.29 (1.09-1.53)
Gestational hypertension	150	0.64	1.36 (1.16-1.59)	1.32 (1.12-1.55)
**Hypermetropia**				
No maternal HDP	11201	0.33	1 [Reference]	1 [Reference]
Maternal HDP				
Overall	707	0.52	1.54 (1.42-1.66)	1.41 (1.30-1.52)
Preeclampsia or eclampsia				
Overall	485	0.50	1.51 (1.38-1.65)	1.43 (1.31-1.57)
Preeclampsia	481	0.50	1.51 (1.38-1.66)	1.44 (1.31-1.57)
Moderate	308	0.43	1.29 (1.15-1.44)	1.27 (1.13-1.42)
Severe	119	0.76	2.23 (1.86-2.67)	1.91 (1.59-2.29)
HELLP syndrome	19	1.05	2.81 (1.79-4.41)	1.82 (1.16-2.86)
Unspecified	35	0.59	1.80 (1.29-2.51)	1.82 (1.30-2.53)
Eclampsia	NR^b^	0.42	1.27 (0.48-3.39)	1.17 (0.44-3.13)
Hypertension				
Overall	222	0.56	1.60 (1.40-1.83)	1.36 (1.19-1.55)
Pregestational hypertension	108	0.68	1.85 (1.53-2.24)	1.37 (1.13-1.66)
Gestational hypertension	114	0.48	1.42 (1.18-1.71)	1.35 (1.12-1.62)
**Myopia**				
No maternal HDP	2843	0.08	1 [Reference]	1 [Reference]
Maternal HDP				
Overall	150	0.11	1.32 (1.12-1.56)	1.30 (1.10-1.53)
Preeclampsia or eclampsia				
Overall	114	0.12	1.40 (1.16-1.69)	1.36 (1.13-1.65)
Preeclampsia	113	0.12	1.40 (1.16-1.69)	1.37 (1.13-1.65)
Moderate	69	0.10	1.13 (0.89-1.43)	1.13 (0.89-1.43)
Severe	27	0.17	2.05 (1.40-3.00)	1.84 (1.26-2.70)
HELLP syndrome	7	0.38	5.13 (2.45-10.75)	4.47 (2.13-9.40)
Unspecified	10	0.17	1.96 (1.05-3.65)	1.93 (1.03-3.58)
Eclampsia	NR^b^	0.11	1.25 (0.18-8.91)	1.14 (0.16-8.11)
Hypertension				
Overall	36	0.09	1.13 (0.81-1.57)	1.13 (0.81-1.57)
Pregestational hypertension	13	0.08	1.05 (0.61-1.81)	1.00 (0.58-1.73)
Gestational hypertension	23	0.10	1.18 (0.78-1.78)	1.22 (0.81-1.83)
**Astigmatism**				
No maternal HDP	2513	0.07	1 [Reference]	1 [Reference]
Maternal HDP				
Overall	148	0.11	1.45 (1.23-1.71)	1.45 (1.22-1.71)
Preeclampsia or eclampsia				
Overall	108	0.11	1.50 (1.23-1.81)	1.53 (1.26-1.86)
Preeclampsia	108	0.11	1.51 (1.25-1.83)	1.55 (1.27-1.88)
Moderate	64	0.09	1.19 (0.93-1.52)	1.26 (0.98-1.62)
Severe	28	0.18	2.37 (1.63-3.43)	2.15 (1.48-3.12)
HELLP syndrome	NR^b^	0.22	2.89 (1.08-7.70)	2.15 (0.81-5.74)
Unspecified	12	0.20	2.71 (1.54-4.78)	2.84 (1.61-5.01)
Eclampsia	NR^b^	NR^b^	NR^b^	NR^b^
Hypertension				
Overall	40	0.10	1.34 (0.98-1.83)	1.26 (0.92-1.72)
Pregestational hypertension	18	0.11	1.48 (0.93-2.35)	1.20 (0.75-1.91)
Gestational hypertension	22	0.09	1.25 (0.82-1.90)	1.31 (0.86-2.00)
**Other types of RE**				
No maternal HDP	1436	0.04	1 [Reference]	1 [Reference]
Maternal HDP				
Overall	98	0.07	1.67 (1.36-2.04)	1.60 (1.30-1.96)
Preeclampsia or eclampsia				
Overall	71	0.07	1.72 (1.36-2.18)	1.69 (1.33-2.15)
Preeclampsia	71	0.07	1.74 (1.37-2.21)	1.71 (1.34-2.18)
Moderate	50	0.07	1.63 (1.23-2.16)	1.68 (1.26-2.23)
Severe	13	0.08	1.90 (1.10-3.29)	1.63 (0.94-2.81)
HELLP syndrome	NR^b^	0.16	3.53 (1.14-10.97)	2.27 (0.73-7.06)
Unspecified	NR^b^	0.08	1.99 (0.83-4.79)	2.10 (0.87-5.05)
Eclampsia	NR^b^	NR^b^	NR^b^	NR^b^
Hypertension				
Overall	27	0.07	1.53 (1.05-2.25)	1.39 (0.95-2.03)
Pregestational hypertension	13	0.08	1.77 (1.02-3.05)	1.38 (0.80-2.39)
Gestational hypertension	14	0.06	1.37 (0.81-2.31)	1.39 (0.82-2.36)

Abbreviations: HDP, hypertensive disorder of pregnancy; HELLP, hemolysis, elevated liver enzyme level, and low platelet count; NR, not reported; RE, refractive error.

^a^ Adjusted for calendar year, sex, singleton, parity, maternal age, maternal smoking, maternal cohabitation, maternal country of origin, maternal residence, maternal education duration, maternal income at birth, maternal prepregnancy body mass index, and parental RE before childbirth.

^b^ Not allowed to report when there were fewer than 6 cases due to data protection in Denmark.

**Figure 2.**
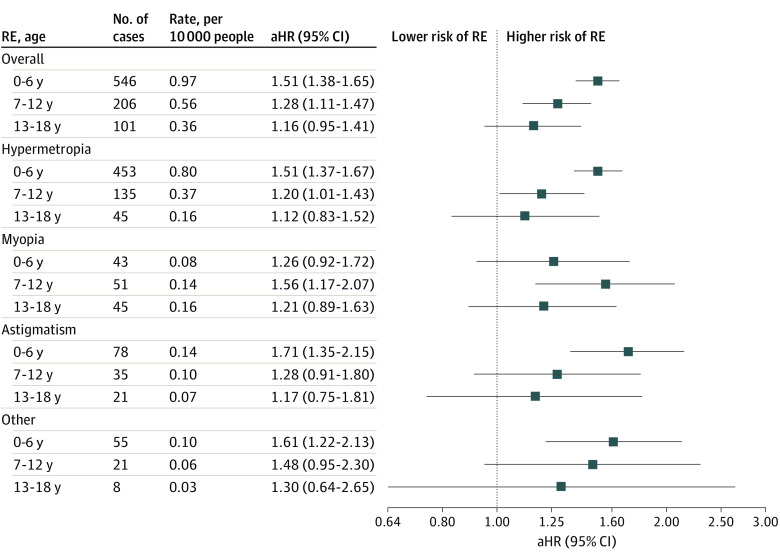
Association Between Maternal Hypertensive Disorder of Pregnancy and Overall and Specific High Refractive Error (RE) in Offspring by Offspring Age Data are from analysis adjusted for calendar year, sex, singleton, parity, maternal age, maternal smoking, maternal cohabitation, maternal country of origin, maternal residence, maternal education duration, maternal income at birth, maternal prepregnancy body mass index, and parental RE before childbirth. aHR indicates adjusted hazard ratio.

The increased risk of high RE was observed for offspring younger than 6 years (HR, 1.51; 95% CI, 1.38-1.65) and aged 7 to 12 years (HR, 1.28; 95% CI, 1.11-1.47) and 13 to 18 years (HR, 1.16; 95% CI, 0.95-1.41), but the difference was not significant for the oldest group. Similar patterns were also observed for type-specific high REs (Figure 2).

Compared with offspring exposed to late-onset preeclampsia (HR, 1.20; 95% CI, 1.07-1.34), offspring prenatally exposed to early-onset preeclampsia (HR, 1.80; 95% CI, 1.60-2.02) had a higher estimated risk of high RE. When considering both the timing of diagnosis and severity of maternal preeclampsia, the highest risk was observed in offspring prenatally exposed to early-onset and severe preeclampsia (HR, 2.59; 95% CI, 2.17-3.08) (Table 3).

**Table 3.** Risk of Overall High Refractive Error in Offspring According to the Timing and Severity of Maternal Preeclampsia^a^

	Cases, No.	Rate, per 1000 person-years	Hazard ratio (95% CI)	
			Crude	Adjusted^b^
Timing of preeclampsia				
Late onset	321	0.53	1.15 (1.03-1.28)	1.20 (1.07-1.34)
Early onset	293	1.03	2.17 (1.93-2.44)	1.80 (1.60-2.02)
Severity of preeclampsia				
Moderate	426	0.59	1.28 (1.16-1.41)	1.28 (1.16-1.41)
Severe and HELLP	188	1.07	2.29 (1.98-2.64)	1.93 (1.67-2.23)
Interaction for timing and severity of preeclampsia				
Late onset × moderate	261	0.50	1.10 (0.98-1.25)	1.18 (1.05-1.34)
Late onset × severe or HELLP	60	0.65	1.39 (1.08-1.79)	1.25 (0.97-1.62)
Early onset × moderate	165	0.82	1.72 (1.47-2.00)	1.46 (1.25-1.70)
Early onset × severe or HELLP	128	1.55	3.27 (2.75-3.90)	2.59 (2.17-3.08)

Abbreviation: HELLP, hemolysis, elevated liver enzyme level, and low platelet count.

^a^ Includes moderate preeclampsia, severe preeclampsia, and HELLP syndrome.

^b^ Adjusted for calendar year, sex, singleton, parity, maternal age, maternal smoking, maternal cohabitation, maternal country of origin, maternal residence, maternal education duration, maternal income at birth, maternal prepregnancy body mass index, and parental refractive error before childbirth.

The sibling-matched analysis also yielded an increased risk of overall high RE in half siblings (HR, 1.21; 95% CI, 1.05-1.39) and full siblings (HR, 1.15; 95% CI, 0.99-1.34), but the difference was not significant for the latter (eTable 4 in Supplement 1). Analyses stratified by baseline characteristics revealed almost identical findings across different strata (eTable 5 in Supplement 1). There was no association observed when using paternal hypertension before pregnancy as a control exposure (HR, 1.16; 95% CI, 0.94-1.42) (eTable 6 in Supplement 1). Analyses stratified by small for gestational age or not indicated that the HRs for the associations between maternal HDP and offspring high RE were similar in the offspring with birth weight below the 10th percentile (HR, 1.32; 95% CI, 1.14-1.53) and in those with birth weight above the 10th percentile (HR, 1.36; 95% CI, 1.26-1.46) (eTable 7 in Supplement 1). Results from the subgroup analyses were similar to the main analysis when additionally adjusting for paternal hypertension; restricting to offspring born after 1991, 1994, 2004; using a complete cases analysis; or restricting to offspring born to primipara (eTable 8 in Supplement 1). Analysis restricting to term-born individuals also yielded similar results (eTable 9 in Supplement 1).

## Discussion

In this large population-based cohort study, we found that offspring born to mothers with HDP had increased risk of overall and type-specific high REs, including hypermetropia, myopia, and astigmatism, in their childhood and adolescence. The increased risk of high RE was found among offspring aged 12 years or younger. The highest risk of high RE was observed in offspring born to mothers with early-onset and severe preeclampsia.

There might be several possible explanations for the observed associations. First, women with HDP have changes in the serum level of circulation antiangiogenic factors, such as higher soluble fms-like tyrosine kinase (sFlt-1) and lower placental growth factor (PIGF) levels.^30^ The increased sFlt-1 and decreased PIGF levels during pregnancy may have persistent influence on ocular microvascular structure and ocular blood flow in offspring, thereby affecting retinal development and leading to morphologic changes.^31^ Previous studies have also shown that HDP can lead to changes in retinal microvasculature in offspring, including narrower retinal arteriolar and venular calibers.^21^ Second, HDP can lead to excessive oxidative stress and inflammation during pregnancy and affect multiple organ systems by activating different pathophysiological mechanisms.^32^ In animal models, exposure to oxidative stress causes degeneration of photoreceptors and other cells of the neural retina in macaques.^33^ This finding supported that HDP may damage the retina of progeny and affect refractive development in offspring through oxidative stress resulting in short- and long-term refractive errors.^5,34^ Third, individuals born to a preeclamptic pregnancy would have increased risk of preterm birth due to placental dysfunction and may therefore have a higher risk of subsequent RE.^35,36^

Empirical evidence is lacking for the association of maternal HDP with overall high RE and specific types in offspring in childhood and adolescence. The proportion of maternal hypertension was higher in children with congenital myopia (11 of 38 cases) than in the control group with other visual defects (2 of 39 cases) in a small case-control study.^19^ Maternal HDP or its subtypes may be associated with other abnormal visual conditions and eye diseases in offspring, such as retinopathy of prematurity,^20^ narrower retinal arteriolar and venular caliber in 6-year-old children,^21^ thinner macular ganglion cell-inner plexiform layer and peripapillary retinal nerve fiber layer thickness in preschool-age offspring,^22^ larger optic cup areas in 5-year-old preterm children,^23^ and amblyopia in young adults aged 20 years^24^; these findings suggest that maternal HDP has deleterious consequences for offspring visual development. Our study is, to our knowledge, the first population-based cohort study to show that children of mothers with HDP had a higher risk of overall and type-specific high REs, including hypermetropia, myopia, and astigmatism. The risk increased with the severity of the preeclampsia. Hypertensive disorder of pregnancy and diabetes are 2 of the most common cardiometabolic disorders that occur during pregnancy.^13^ Consistent with our findings, previous studies^5,6,7,8^ have suggested that maternal diabetes during pregnancy is associated with an increased risk of overall high RE, hyperopia, and myopia. Two prospective studies in Denmark^5^ (N = 2 470 580) and China^6^ (N = 301) found that offspring exposed to maternal diabetes during pregnancy also had a higher risk of astigmatism, although the difference was not significant in the Chinese study (HR, 1.58 [95% CI, 1.29-1.92] for the Danish study; odds ratio, 2.48 [95% CI, 0.97-6.32] for the Chinese study). The similarity in disease risk due to maternal diabetes and maternal HDP for overall high RE may suggest a shared pathological process between maternal diabetes and maternal HDP in refractive development in offspring.^13,37^ However, a study^7^ of children in the ophthalmological outpatient clinic of a pediatric hospital in Barcelona (N = 350) found that the prevalence of astigmatism was similar among children exposed (4 of 229 children) and unexposed (1 of 121 children) to maternal diabetes during pregnancy, and another cross-sectional study^8^ of 33 neonates in Turkey also found no significant difference in astigmatism between the maternal diabetic group (mean [SD] cylindrical refraction, 0.5 [1.1] diopters) and the unexposed group (mean [SD] cylindrical refraction, 0.6 [0.9] diopters. The inconsistent results in astigmatism between these 2 studies^7,8^ and ours may be because of differences in study design, sample size, and country of origins of the study population.

Our study found increased risk of high RE for offspring in different age groups, which was consistent with a Danish study that found maternal diabetes during pregnancy was associated with a higher risk of high RE in offspring in different age groups.^5^ We also observed that the risk of hyperopia was highest in the group younger than 6 years, which is probably because hyperopia is the most common RE in childhood,^38^ as individuals’ eyes are predominantly hyperopic from birth and refractive change and axial growth after birth undergo a process of emmetropization characterized by a reduction in RE over time.^12^ However, we also found an increased risk of hyperopia at the ages of 7 to 12 years, suggesting that HDP may have an independent and persistent association with refractive conditions in offspring. In addition, the risk of myopia was slightly greater among offspring aged 7 to 12 years, which may be due to the accompanying academic burden and worse eye habits.^39^ It is important to note that maternal HDP was associated with increased risk of all types of RE in offspring in our study, although the HRs were slightly different among different subtypes. This suggests that maternal HDP may be associated with short- and long-term abnormal refractive regulation through adverse intrauterine environment and with increased risk of high RE from birth to adolescence.^24,37^

We observed that early-onset and severe maternal preeclampsia was associated with a higher risk of high RE in childhood and adolescence. Previous studies also suggested that offspring of mothers with early-onset and severe preeclampsia would be at higher risk of developing cardiovascular disease, impaired fetal cardiac function, higher blood pressure, neurodevelopmental disorders, and premature death.^16,17,18,40,41,42,43^ It is possible that pathological conditions, such as placental oxidative stress, immune and inflammation response, placental malperfusion and metabolic abnormality, abnormal blood pressure, and urine protein levels, were worse in early-onset and severe preeclampsia, which would contribute to the abnormal refractive development and regulation later in life.^32,34,44,45^ These findings suggest that offspring exposed to early-onset and severe preeclampsia should be given intense attention to ophthalmic and especially refractive examinations.

### Strengths and Limitations

Our study has the strengths of high-quality follow-up data with large sample size including nearly all live births in Denmark, which would minimize recall and selection bias and allowed us to explore the influence of specific types of HDP and subtypes of high RE. In addition, the validity of records for diagnoses in Danish registers was high, which to some extent, reduced the possibility of substantial misclassification of maternal HDP and offspring REs.^46^

Several limitations should be noted. First, we cannot rule out the possibility of residual confounding as some important factors, such as maternal genetic factors, alcohol consumption, diet and nutrition, outdoor activity, and other lifestyle factors, are not available in our study. However, the results of the sibling analysis were similar to those of the main analysis, and no association between paternal hypertension (as control exposure) and offspring REs was observed, suggesting that the observed association between maternal HDP and high RE in offspring might not be entirely attributable to genetic or familial factors. Second, our study was based on register data from Denmark, and not all REs were recorded in the Danish National Patient Register; only offspring with severe RE who received intensive ophthalmic examination were recorded in the register. Therefore, we were unable to estimate the overall association between maternal HDP and complete coverage of RE. However, our study provided the best available evidence from Denmark thus far, and future research on maternal HDP and complete diagnosis of RE are warranted. Third, Denmark has universal health coverage for public health services, which may limit the generalizability of our results.

## Conclusions

In this cohort study, offspring of mothers with HDP had increased risk of overall and type-specific high REs, including hypermetropia, myopia, and astigmatism, in their childhood and adolescence. These findings suggest that early screening of ophthalmic RE should be recommended for offspring prenatally exposed to maternal HDP, especially those of mothers with severe and early-onset preeclampsia.

## Notes

Supplement 1. **eAppendix 1.** Detailed description of multiple imputation methods **eTable 1.** Detailed descriptions of registers used in this study **eTable 2.** Exposure classification of hypertensive disorders of pregnancy from the International Classification of Diseases, the 8th and 10th versions (ICD-8 and ICD-10) **eTable 3.** Outcome classification of refractive errors from the International Classification of Diseases, the 8th and 10th versions (ICD-8 and ICD-10) **eTable 4.** Associations between maternal HDP and high REs in offspring of sibling pairs **eTable 5.** Associations between maternal HDP and high REs according to baseline characteristics **eTable 6.** Associations between paternal hypertension before pregnancy and high RE in offspring **eTable 7.** Associations between maternal HDP and high RE in offspring according to SGA **eTable 8.** Subanalyses of the association between maternal HDP and high REs in offspring **eTable 9.** Association of maternal HDP with offspring high RE restricting to term-born individuals **eFigure 1.** Causal diagram showing selection of covariates for confounding control **eFigure 2.** The log-minus-log survival curve for maternal HDP and offspring overall high RE

Supplement 2.
Data Sharing Statement
