# Supplementary material for: Evaluation of Hypertensive Disorder of Pregnancy and High Refractive Error in Offspring During Childhood and Adolescence
Source: JAMA Netw Open. 2023 Apr 18;6(4):e238694. doi: 10.1001/jamanetworkopen.2023.8694 (PMC10114077; doi:10.1001/jamanetworkopen.2023.8694)
Supplement: Supplement 1. — eAppendix 1. Detailed description of multiple imputation methods eTable 1. Detailed descriptions of registers used in this study eTable 2. Exposure classification of hypertensive disorders of pregnancy from the International Classification of Diseases, the 8th and 10th versions (ICD-8 and ICD-10) eTable 3. Outcome classification of refractive errors from the International Classification of Diseases, the 8th and 10th versions (ICD-8 and ICD-10) eTable 4. Associations between maternal HDP and high REs in offspring of sibling pairs eTable 5. Associations between maternal HDP and high REs according to baseline characteristics eTable 6. Associations between paternal hypertension before pregnancy and high RE in offspring eTable 7. Associations between maternal HDP and high RE in offspring according to SGA eTable 8. Subanalyses of the association between maternal HDP and high REs in offspring eTable 9. Association of maternal HDP with offspring high RE restricting to term-born individuals eFigure 1. Causal diagram showing selection of covariates for confounding control eFigure 2. The log-minus-log survival curve for maternal HDP and offspring overall high RE [file jamanetwopen-e238694-s001.pdf]

Supplemental Online Content

Li M, Huang C, Yang W, et al. Evaluation of hypertensive disorder of pregnancy and high refractive error in offspring during childhood and adolescence. *JAMA Netw Open.* 2023;6(4):e238694. doi:10.1001/jamanetworkopen.2023.8694

eAppendix 1. Detailed description of multiple imputation methods

eTable 1. Detailed descriptions of registers used in this study

eTable 2. Exposure classification of hypertensive disorders of pregnancy from the International Classification of Diseases, the 8th and 10th versions (ICD-8 and ICD-10)

eTable 3. Outcome classification of refractive errors from the International Classification of Diseases, the 8th and 10th versions (ICD-8 and ICD-10)

eTable 4. Associations between maternal HDP and high REs in offspring of sibling pairs

eTable 5. Associations between maternal HDP and high REs according to baseline characteristics

eTable 6. Associations between paternal hypertension before pregnancy and high RE in offspring

eTable 7. Associations between maternal HDP and high RE in offspring according to SGA

eTable 8. Subanalyses of the association between maternal HDP and high REs in offspring

eTable 9. Association of maternal HDP with offspring high RE restricting to term-born individuals

eFigure 1. Causal diagram showing selection of covariates for confounding control

eFigure 2. The log-minus-log survival curve for maternal HDP and offspring overall high RE

This supplemental material has been provided by the authors to give readers additional information about their work.

eAppendix. Detailed description of multiple imputation methods

Multiple imputation methods imputed with 10 replications was used to deal with missing values in this study.[1] We imputed missing variables using the fully conditional specification (FCS) method in which logistic regression was used for categorical variables and predictive mean matching for continuous variables. The imputation model included the following variables: maternal HDP (yes, no), offspring high RE (yes, no), sex (male, female), singleton (yes, no), parity (1, 2, or ≥3 children), the birth year of the child (1978-1980, 5-year intervals during 1981-2015, and 2016-2018), maternal age (<20, 20-24, 25-29, 30-34, ⩾35 years), maternal smoking during pregnancy (yes, no), maternal cohabitation (single, cohabitating), maternal residence (Copenhagen, big cities ≥ 100 000 inhabitants, others), maternal country of origin (Denmark, non-Denmark), maternal education before pregnancy (0-9 years, 10-14 years, ≥15 years), maternal income at birth (no income, three tertiles), maternal pre-pregnancy body mass index (BMI) (<18.5 kg/m2, 18.5-24.9 kg/m2, 25.0-29.9 kg/m2, ≥30.0 kg/m2), maternal history of RE and paternal history of RE before childbirth (yes, no). As the data on maternal smoking was available since 1991 and data on maternal pre-pregnancy BMI was available since 2004, the imputation model for maternal smoking and maternal pre-pregnancy BMI was only restricted to offspring born since 1991 and 2004, respectively.

eTable 1. Detailed descriptions of registers used in this study [a]

Register	Description
The Danish Civil Registration System (CRS)[2]	The Danish Civil Registration System (CRS) was established in 1968 and collected information on the date of birth, emigration, immigration, sex, and other vital status on a daily basis.
The Danish Medical Birth Registry (MBR)[3]	The Danish Medical Birth Registry (MBR) includes CPR number of new births, mothers, and fathers, as well as information on the date of birth, birth characteristics such as sex, birth weight, gestational age, maternal characteristics such as maternal pre-pregnancy body mass index, maternal smoking during pregnancy. MBR was established in 1968 and has been computerized since 1973.
The Danish National Patient Register (DNPR)[4]	The Danish National Patient Register (DNPR) was established in 1977 and collected data from inpatient, outpatient, and emergency hospital records (each visit for one record in the register). Information includes the date of contact, primary and secondary discharge diagnoses, date and procedures of surgery, certain treatments in hospitals, and other hospital-related information. Diagnoses are classified according to the ICD-8 codes (*International Classification of Disease* codes, 8th revision) and ICD-10 codes thereafter.
The Danish Registry of Causes of Death[5]	The Danish Registry of Causes of Death has been computerized since 1970, and non-electronic data on deaths has been collected since 1924. This system has been collecting the date of death, immediate cause, underlying cause and contributory causes of death by law in Denmark. The ICD-8 was used to classify the causes of death during 1970-1993, and ICD-10 from 1994.
The Danish Integrated Database for Longitudinal Labour Market Research[6]	The Danish Integrated Database for Longitudinal Labour Market Research[5] was established in 1981 and contains labour market and socioeconomic data.

[a] Denmark has been providing public welfare, including universal health care, education, student assistance, disability pensions, and unemployment insurance through tax-funded services. Denmark's population-based health databases regularly collect high-quality data, including individual medical data. All live births and new residents in Denmark have been assigned a unique 10-digit individual personal identification number (Central Personal Register number, CPR) since the 1960s. The CPR number links information across different national registers.

© 2023 Li M et al. *JAMA Network Open.*

eTable 2. Exposure classification of hypertensive disorders of pregnancy from the International Classification of Diseases, the 8th and 10th versions (ICD-8 and ICD-10)

	ICD-8	ICD-10
**Pre-eclampsia**		
Moderate pre-eclampsia	63703	O14.0
Severe pre-eclampsia	63704	O14.1
HELLP syndrome		O14.2
Unspecified pre-eclampsia	63709	O14.9
**Eclampsia**	63719	O15
**Gestational hypertension**		
Gestational (pregnancy-induced) hypertension	63700	O13
Unspecified maternal hypertension	76029	O16
**Pre-gestational hypertension**		
Essential hypertension	40009	I10
Hypertensive heart disease	40019<br>40029	I11
Hypertensive renal disease	40039	I12
Hypertensive heart and renal disease	40099	I13
Secondary hypertension	40199	I15
Pre-existing hypertension complicating pregnancy		O10
Pre-eclampsia superimposed on chronic hypertension		O11

© 2023 Li M et al. *JAMA Network Open.*

eTable 3. Outcome classification of refractive errors from the International Classification of Diseases, the 8th and 10th versions (ICD-8 and ICD-10)

Outcome	ICD-8	ICD-10
**Overall refractive error**	37000;37001;37002;37003;37004;37008;37009	H52.0;H52.1;H52.2;H52.3; H52.5; H52.6; H52.7
**Specific types of refractive error**		
Hypermetropia	37001	H52.0
Myopia	37002	H52.1
Astigmatism	37000	H52.2
Other types of refractive error	37003;37004;37008;37009	H52.3; H52.5; H52.6; H52.7

© 2023 Li M et al. *JAMA Network Open.*

eTable 4. Associations between maternal HDP and high REs in offspring of sibling pairs

Sibling design	Exposure	No. of cases	Rate ($1/10^3$)	cHR (95%CI)	aHR [b] (95%CI)
**Half-sibling** [a]	No maternal HDP	13549	0.47	1.0(reference)	1.0(reference)
	Maternal HDP	772	0.70	1.21(1.06-1.39)	1.21(1.05-1.39)
**Full-sibling** [a]	No maternal HDP	11883	0.46	1.0(reference)	1.0(reference)
	Maternal HDP	672	0.68	1.17(1.02-1.36)	1.15(0.99-1.34)

Abbreviations: HDP, hypertensive disorders of pregnancy; RE, refractive error; cHR, crude hazard ratio; aHR, adjusted hazard ratio.

[a] Half-sibling: sibling pairs of offspring born to the same mother; full-sibling: sibling pairs of offspring born to the same father and mother.

[b] Adjusted for calendar year, sex, singleton, parity, maternal age, maternal smoking, maternal cohabitation, maternal country of origin, maternal residence, maternal education, maternal income at birth, maternal pre-pregnancy BMI, and parental RE before childbirth.

© 2023 Li M et al. *JAMA Network Open.*

eTable 5. Associations between maternal HDP and high REs according to baseline characteristics

Characteristics	Maternal HDP			Hypertension			Pre-eclampsia or eclampsia		
	No. of cases	Rate (1/10 [3])	HR (95% CI) [a]	No. of cases	Rate (1/10 [3])	HR (95% CI) [a]	No. of cases	Rate (1/10 [3])	HR (95% CI) [a]
**Singleton**									
No	115	1.20	1.24(1.02-1.51)	22	1.34	1.28(0.84-1.96)	93	1.17	1.23(0.99-1.52)
Yes	831	0.66	1.41(1.31-1.51)	258	0.68	1.31(1.15-1.48)	573	0.65	1.46(1.34-1.59)
**Gender**									
Boy	494	0.70	1.41(1.29-1.55)	145	0.71	1.31(1.11-1.55)	349	0.70	1.46(1.31-1.63)
Girl	452	0.69	1.37(1.25-1.51)	135	0.71	1.30(1.09-1.54)	317	0.69	1.41(1.26-1.58)
**Maternal parity**									
1	583	0.72	1.38(1.27-1.51)	140	0.75	1.33(1.13-1.58)	443	0.71	1.40(1.27-1.54)
2	221	0.61	1.29(1.13-1.48)	83	0.62	1.19(0.96-1.48)	138	0.61	1.36(1.15-1.61)
≥3	142	0.76	1.58(1.33-1.87)	57	0.76	1.42(1.09-1.85)	85	0.76	1.7(1.37-2.11)
**Maternal age at childbirth (years)**									
<20 [b]	20	0.53	1.23(0.78-1.93)	-	-	-	20	0.60	1.40(0.89-2.21)
20-24	133	0.50	1.28(1.07-1.52)	26	0.52	1.25(0.85-1.85)	107	0.49	1.28(1.05-1.56)
25-29	328	0.69	1.44(1.28-1.61)	87	0.70	1.39(1.12-1.72)	241	0.69	1.46(1.28-1.66)
30-34	288	0.79	1.46(1.30-1.65)	99	0.77	1.38(1.13-1.69)	189	0.80	1.51(1.30-1.75)
35+	177	0.84	1.32(1.13-1.55)	68	0.78	1.19(0.93-1.51)	109	0.88	1.43(1.18-1.74)
**Maternal smoking during pregnancy**									
No	615	0.89	1.43(1.32-1.56)	195	0.83	1.33(1.15-1.53)	420	0.92	1.49(1.35-1.64)
Yes	161	1.20	1.56(1.33-1.83)	48	1.17	1.43(1.08-1.91)	113	1.22	1.62(1.34-1.95)
Unknown	170	0.32	1.15(0.99-1.34)	37	0.31	1.10(0.80-1.52)	133	0.32	1.17(0.98-1.39)
**Maternal education at childbirth, years**									
0-9	260	0.65	1.37(1.21-1.55)	62	0.65	1.21(0.94-1.55)	198	0.65	1.43(1.24-1.65)
10-14	409	0.66	1.33(1.20-1.47)	113	0.63	1.14(0.95-1.38)	296	0.68	1.41(1.26-1.59)
15+	266	0.81	1.50(1.32-1.70)	100	0.87	1.58(1.29-1.92)	166	0.77	1.46(1.25-1.71)
**Maternal cohabitation at childbirth**									
No	497	0.76	1.44(1.32-1.58)	143	0.83	1.42(1.2-1.68)	354	0.74	1.45(1.30-1.62)
Yes	449	0.63	1.35(1.22-1.48)	137	0.62	1.20(1.01-1.42)	312	0.64	1.42(1.27-1.59)
**Maternal residence at childbirth**									
Copenhagen	85	0.67	1.35(1.08-1.68)	24	0.71	1.36(0.91-2.04)	61	0.66	1.34(1.04-1.74)

© 2023 Li M et al. *JAMA Network Open.*

Big cities≥100 000 inhabitants	145	0.81	1.38(1.16-1.64)	52	0.83	1.52(1.15-2.00)	93	0.79	1.31(1.06-1.62)
Others	716	0.68	1.40(1.30-1.51)	204	0.68	1.26(1.10-1.45)	512	0.68	1.46(1.34-1.60)
**Maternal country of origin**									
Denmark	70	0.89	1.47(1.15-1.87)	17	0.79	1.22(0.76-1.97)	53	0.93	1.57(1.19-2.07)
Non-Denmark	876	0.69	1.39(1.29-1.49)	263	0.71	1.31(1.16-1.48)	613	0.68	1.42(1.31-1.55)
**Maternal income**									
No income	193	0.96	1.72(1.49-1.99)	59	1.02	1.67(1.29-2.16)	134	0.93	1.74(1.46-2.08)
Less than the lower tertiles	242	0.68	1.29(1.13-1.47)	55	0.59	1.02(0.78-1.33)	187	0.72	1.40(1.21-1.63)
Lower and higher tertiles	253	0.68	1.30(1.14-1.48)	79	0.69	1.23(0.99-1.54)	174	0.67	1.33(1.14-1.55)
More than the higher tertiles	250	0.70	1.42(1.25-1.61)	85	0.76	1.45(1.16-1.79)	165	0.68	1.40(1.20-1.64)
**Pre-pregnancy maternal BMI**									
<18.5 [b]	8	1.17	1.34(0.66-2.72)	-	-	-	-	-	-
18.5-24.9	167	1.14	1.55(1.32-1.81)	48	0.78	1.14(0.86-1.52)	119	1.40	1.81(1.51-2.19)
25.0-29.9	89	1.12	1.35(1.09-1.68)	37	1.12	1.44(1.04-2.00)	52	1.12	1.29(0.98-1.72)
>=30.0	117	1.49	1.59(1.30-1.95)	54	1.52	1.73(1.31-2.30)	63	1.46	1.49(1.14-1.94)
Unknown	565	0.54	1.33(1.22-1.44)	139	0.53	1.25(1.05-1.47)	426	0.54	1.36(1.23-1.50)
**Maternal RE history before childbirth**									
No	939	0.69	1.40(1.31-1.49)	277	0.70	1.30(1.16-1.47)	662	0.69	1.44(1.33-1.56)
Yes [b]	7	1.76	1.36(0.62-2.97)	-	-	-	-	-	-
**Paternal RE history before childbirth**									
No	932	0.69	1.40(1.31-1.49)	276	0.71	1.31(1.16-1.47)	656	0.69	1.44(1.33-1.56)
Yes [b]	-	-	-	-	-	-	-	-	-

Abbreviations: HDP, hypertensive disorder of pregnancy; RE, refractive error; cHR, crude hazard ratio; aHR, adjusted hazard ratio.

[a] Adjusted for calendar year, sex, singleton, parity, maternal age, maternal smoking, maternal cohabitation, maternal country of origin, maternal residence, maternal education, maternal income at birth, maternal pre-pregnancy BMI, and parental RE before childbirth.

[b] <6 cases are not allowed to report due to data protection in Denmark.

© 2023 Li M et al. *JAMA Network Open.*

eTable 6. Associations between paternal hypertension before pregnancy and high RE in offspring

Exposure	No. of cases	Rate (1/10^3)	cHR (95%CI)	aHR [a] (95%CI)
No paternal hypertension	16266	0.47	1.0(reference)	1.0(reference)
Paternal hypertension	89	0.70	1.44(1.17-1.77)	1.16(0.94-1.42)

Abbreviations: RE, refractive error; cHR, crude hazard ratio; aHR, adjusted hazard ratio.

[a] Adjusted for calendar year, sex, singleton, parity, maternal age, maternal smoking, maternal cohabitation, maternal country of origin, maternal residence, maternal education, maternal income at birth, maternal pre-pregnancy BMI, and parental RE before childbirth.

© 2023 Li M et al. *JAMA Network Open.*

eTable 7. Associations between maternal HDP and high RE in offspring according to SGA

SGA	Exposure	No. of cases	Rate ($1/10^3$)	cHR (95%CI)	aHR [a] (95%CI)
**Birthweight below the 10th percentile**	No maternal HDP	2156	0.73	1.0(reference)	1.0(reference)
	Maternal HDP	199	1.00	1.35(1.17-1.57)	1.32(1.14-1.53)
**Otherwise**	No maternal HDP	13188	0.44	1.0(reference)	1.0(reference)
	Maternal HDP	739	0.64	1.46(1.36-1.57)	1.36(1.26-1.46)

Abbreviations: HDP, hypertensive disorder of pregnancy; RE, refractive error; SGA, small for gestational age; cHR, crude hazard ratio; aHR, adjusted hazard ratio.

[a] Adjusted for calendar year, sex, singleton, parity, maternal age, maternal smoking, maternal cohabitation, maternal country of origin, maternal residence, maternal education, maternal income at birth, maternal pre-pregnancy BMI, and parental RE before childbirth.

© 2023 Li M et al. *JAMA Network Open.*

eTable 8. Subanalyses of the association between maternal HDP and high REs in offspring

	Exposure	No. of cases	Rate (1/10^3)	cHR (95%CI)	aHR [a] (95%CI)
**Additional adjustment for paternal hypertension**	**No maternal HDP**	15559	0.46	1.0(Reference)	1.0(Reference)
	**Maternal HDP**	946	0.70	1.49(1.40-1.59)	1.39(1.31-1.49)
	Pre-eclampsia or Eclampsia	666	0.69	1.49(1.38-1.61)	1.44(1.33-1.55)
	Pre-eclampsia	661	0.69	1.50(1.38-1.62)	1.44(1.33-1.56)
	Moderate	426	0.59	1.28(1.16-1.41)	1.27(1.16-1.40)
	Severe	160	1.02	2.18(1.86-2.54)	1.89(1.62-2.21)
	HELLP syndrome	28	1.55	3.14(2.16-4.54)	2.15(1.48-3.12)
	Unspecified	47	0.79	1.73(1.30-2.30)	1.74(1.31-2.32)
	Eclampsia [b]	-	0.53	1.14(0.48-2.75)	1.06(0.44-2.54)
	Hypertension	280	0.71	1.48(1.32-1.67)	1.31(1.16-1.47)
	Pregestational hypertension	130	0.81	1.66(1.40-1.98)	1.29(1.09-1.53)
	Gestational hypertension	150	0.64	1.36(1.16-1.59)	1.32(1.12-1.55)
**Offspring born after 1991**	**No maternal HDP**	12285	0.62	1.0(Reference)	1.0(Reference)
	**Maternal HDP**	801	0.97	1.54(1.44-1.66)	1.46(1.36-1.57)
	Pre-eclampsia or Eclampsia	549	1.00	1.62(1.48-1.76)	1.52(1.39-1.65)
	Pre-eclampsia	544	1.00	1.62(1.49-1.76)	1.52(1.39-1.66)
	Moderate	343	0.88	1.43(1.28-1.59)	1.35(1.21-1.50)
	Severe	132	1.26	2.04(1.72-2.42)	1.87(1.58-2.22)
	HELLP syndrome	28	1.55	2.40(1.66-3.48)	2.14(1.48-3.10)
	Unspecified	41	1.30	2.14(1.57-2.90)	2.07(1.52-2.82)
	Eclampsia [b]	-	0.84	1.37(0.57-3.30)	1.30(0.54-3.12)
	Hypertension	252	0.90	1.41(1.24-1.60)	1.35(1.19-1.53)
	Pregestational hypertension	126	0.91	1.40(1.17-1.67)	1.32(1.11-1.58)
	Gestational hypertension	126	0.90	1.42(1.19-1.69)	1.38(1.16-1.65)
**Offspring born after 1994**	**No maternal HDP**	10772	0.65	1.0(Reference)	1.0(Reference)
	**Maternal HDP**	717	1.02	1.54(1.43-1.66)	1.46(1.35-1.58)

© 2023 Li M et al. *JAMA Network Open.*

	Pre-eclampsia or Eclampsia	481	1.07	1.63(1.49-1.79)	1.52(1.38-1.67)
	Pre-eclampsia	477	1.07	1.63(1.49-1.79)	1.52(1.39-1.67)
	Moderate	292	0.92	1.41(1.25-1.58)	1.32(1.17-1.48)
	Severe	125	1.43	2.18(1.82-2.59)	1.99(1.66-2.37)
	HELLP syndrome	28	1.57	2.32(1.60-3.36)	2.15(1.48-3.12)
	Unspecified	32	1.40	2.16(1.52-3.05)	2.03(1.44-2.88)
	Eclampsia [b]	-	0.83	1.27(0.48-3.40)	1.19(0.45-3.17)
	Hypertension	236	0.94	1.38(1.21-1.57)	1.35(1.19-1.54)
	Pregestational hypertension	123	0.94	1.37(1.15-1.64)	1.34(1.12-1.60)
	Gestational hypertension	113	0.94	1.39(1.16-1.68)	1.37(1.14-1.65)
**Offspring born after 2004**	**No maternal HDP**	4303	0.75	1.0(Reference)	1.0(Reference)
	**Maternal HDP**	352	1.22	1.60(1.44-1.79)	1.49(1.34-1.67)
	Pre-eclampsia or Eclampsia	226	1.38	1.82(1.59-2.08)	1.61(1.41-1.85)
	Pre-eclampsia	223	1.38	1.81(1.58-2.07)	1.61(1.40-1.84)
	Moderate	135	1.20	1.58(1.33-1.88)	1.42(1.19-1.68)
	Severe	63	1.86	2.46(1.92-3.15)	2.14(1.67-2.75)
	HELLP syndrome	14	1.57	2.06(1.22-3.48)	1.88(1.11-3.18)
	Unspecified	11	1.54	2.03(1.12-3.66)	1.70(0.94-3.07)
	Eclampsia [b]	-	1.80	2.38(0.77-7.38)	2.15(0.69-6.68)
	Hypertension	126	1.01	1.32(1.11-1.58)	1.32(1.10-1.58)
	Pregestational hypertension	67	0.97	1.28(1.00-1.63)	1.27(1.00-1.62)
	Gestational hypertension	59	1.07	1.38(1.07-1.78)	1.38(1.07-1.79)
**Complete case analysis**	**No maternal HDP**	4369	0.74	1.0(Reference)	1.0(Reference)
	**Maternal HDP**	357	1.20	1.61(1.44-1.79)	1.50(1.35-1.68)
	Pre-eclampsia or Eclampsia	229	1.35	1.81(1.59-2.07)	1.62(1.41-1.85)
	Pre-eclampsia	227	1.35	1.81(1.59-2.07)	1.62(1.41-1.85)
	Moderate	139	1.19	1.59(1.34-1.88)	1.42(1.20-1.69)
	Severe	62	1.84	2.46(1.91-3.16)	2.18(1.70-2.81)
	HELLP syndrome	15	1.65	2.20(1.33-3.65)	2.07(1.25-3.44)
	Unspecified	11	1.38	1.87(1.04-3.39)	1.59(0.88-2.87)
	Eclampsia [b]	-	1.25	1.67(0.42-6.67)	1.50(0.38-6.01)

© 2023 Li M et al. *JAMA Network Open.*

	Hypertension	128	1.01	1.34(1.12-1.59)	1.34(1.12-1.60)
	Pregestational hypertension	67	0.96	1.28(1.00-1.63)	1.29(1.01-1.64)
	Gestational hypertension	61	1.07	1.41(1.09-1.81)	1.40(1.09-1.80)
**Restricting to offspring born to primiparas**	**No maternal HDP**	7413	0.43	1.0(Reference)	1.0(Reference)
	**Maternal HDP**	566	0.65	1.52(1.39-1.65)	1.38(1.27-1.51)
	Pre-eclampsia or Eclampsia	422	0.64	1.49(1.35-1.65)	1.39(1.26-1.54)
	Pre-eclampsia	417	0.64	1.49(1.35-1.65)	1.39(1.26-1.54)
	Moderate	263	0.53	1.25(1.11-1.42)	1.21(1.07-1.37)
	Severe	101	0.92	2.13(1.75-2.60)	1.77(1.45-2.16)
	HELLP syndrome	26	2.08	4.57(3.11-6.71)	2.94(2.00-4.33)
	Unspecified	27	0.67	1.60(1.09-2.33)	1.56(1.07-2.27)
	Eclampsia [b]	-	0.74	1.74(0.72-4.18)	1.50(0.62-3.61)
	Hypertension	144	0.69	1.59(1.35-1.87)	1.36(1.15-1.60)
	Pregestational hypertension	56	0.98	2.19(1.69-2.85)	1.56(1.20-2.03)
	Gestational hypertension	88	0.58	1.35(1.09-1.67)	1.25(1.02-1.55)

Abbreviations: HDP, hypertensive disorder of pregnancy; RE, refractive error; cHR, crude hazard ratio; aHR, adjusted hazard ratio.

[a] Adjusted for calendar year, sex, singleton, parity, maternal age, maternal smoking, maternal cohabitation, maternal country of origin, maternal residence, maternal education, maternal income at birth, maternal pre-pregnancy BMI, and parental RE before childbirth.

[b] <6 cases are not allowed to report due to data protection in Denmark.

© 2023 Li M et al. *JAMA Network Open.*

eTable 9. Association of maternal HDP with offspring high RE restricting to term-born individuals

	No. of cases	Rate ($1/10^3$)	cHR (95%CI)	aHR[a] (95%CI)
Unexposed	13052	0.43	1.0(ref)	1.0(ref)
Exposed	419	0.55	1.29(1.17-1.42)	1.34(1.21-1.47)

Abbreviations: HDP, hypertensive disorder of pregnancy; RE, refractive error; cHR, crude hazard ratio; aHR, adjusted hazard ratio.

[a] Adjusted for calendar year, sex, singleton, parity, maternal age, maternal smoking, maternal cohabitation, maternal country of origin, maternal residence, maternal education, maternal income at birth, maternal pre-pregnancy BMI, and parental RE before childbirth.

© 2023 Li M et al. *JAMA Network Open.*

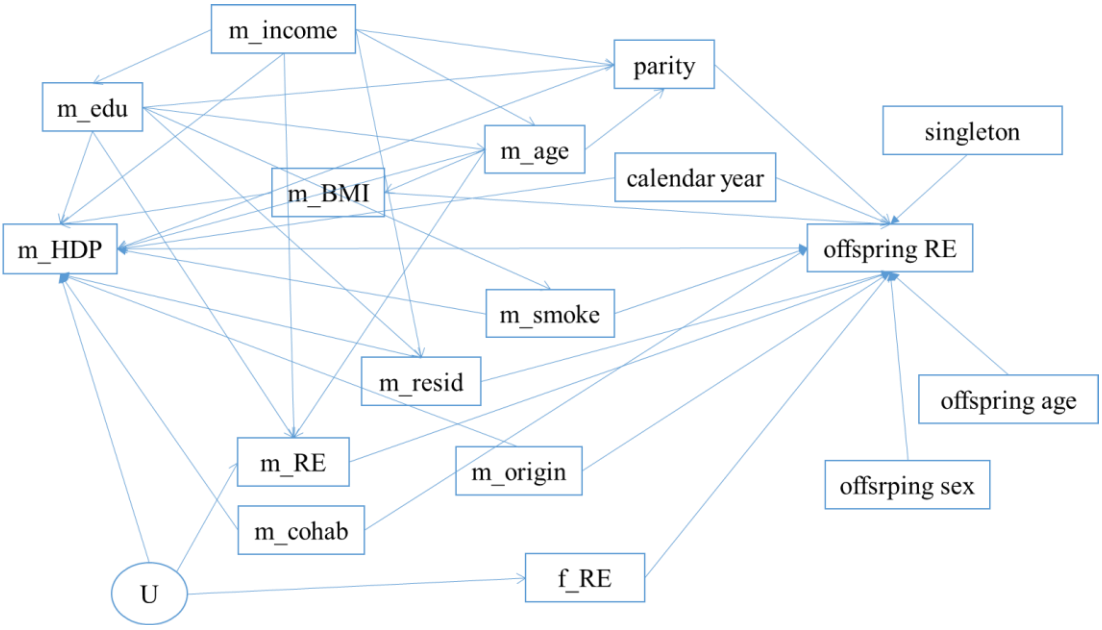

eFigure 1. Causal diagram showing selection of covariates for confounding control [a]

[a] m_HPD: maternal hypertensive disorders of pregnancy, m_age: maternal age, m_smoke: maternal smoking, m_edu: maternal education, m_income: maternal income, m_cohab: maternal cohabitation, m_resid: maternal residence at birth, m_origin: maternal country of origin; m_BMI: maternal BMI, m_RE: maternal history of RE before childbirth, f_RE: paternal history of RE before birth of the child, U: unmeasured variable

© 2023 Li M et al. *JAMA Network Open.*

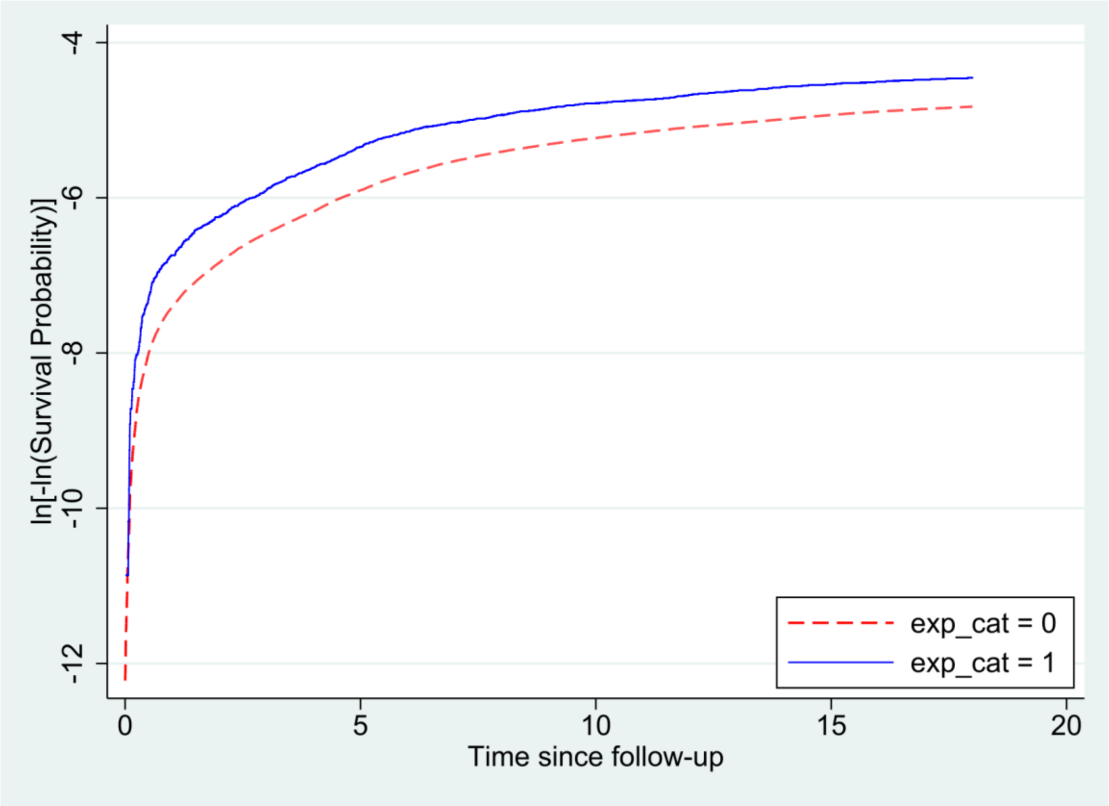

eFigure 2. The log-minus-log survival curve for maternal HDP and offspring overall high RE

© 2023 Li M et al. *JAMA Network Open.*

References

1. Schmidt M, Pedersen L, Sorensen HT. The Danish Civil Registration System as a tool in epidemiology. *European journal of epidemiology.* Aug 2014;29(8):541-549.
2. Schmidt M, Pedersen L, Sorensen HT. The Danish Civil Registration System as a tool in epidemiology. *European journal of epidemiology.* Aug 2014;29(8):541-549.
3. Knudsen LB, Olsen J. The Danish Medical Birth Registry. *Danish medical bulletin.* Jun 1998;45(3):320-323.
4. Lynge E, Sandegaard JL, Rebolj M. The Danish National Patient Register. *Scandinavian journal of public health.* Jul 2011;39(7 Suppl):30-33.
5. Helweg-Larsen K. The Danish Register of Causes of Death. *Scandinavian journal of public health.* Jul 2011;39(7 Suppl):26-29.
6. Petersson F, Baadsgaard M, Thygesen LC. Danish registers on personal labour market affiliation. *Scandinavian journal of public health.* Jul 2011;39(7 Suppl):95-98.

© 2023 Li M et al. *JAMA Network Open.*
