# Supplementary material for: Evaluation of Hypertensive Disorder of Pregnancy and High Refractive Error in Offspring During Childhood and Adolescence
Source: JAMA Netw Open. 2023 Apr 18;6(4):e238694. doi: 10.1001/jamanetworkopen.2023.8694 (PMC10114077; doi:10.1001/jamanetworkopen.2023.8694)
Supplement: Supplement 2. — Data Sharing Statement [file jamanetwopen-e238694-s002.pdf]

Data Sharing Statement

Li. Evaluation of Hypertensive Disorder of Pregnancy and High Refractive Error in Offspring During Childhood and Adolescence. *JAMA Netw Open*. Published April 18, 2023. doi:10.1001/jamanetworkopen.2023.8694

Data
Data available: No

Additional Information
Explanation for why data not available: Due to restrictions related to Danish law and protecting patient privacy, the combined set of data as used in this study can only be made available through a trusted third party, Statistics Denmark (https://www.dst.dk/en/kontakt).
